# Supplementary material for: Novel Flavivirus Antiviral That Targets the Host Nuclear Transport Importin α/β1 Heterodimer
Source: Cells. 2019 Mar 24;8(3):281. doi: 10.3390/cells8030281 (PMC6468590; doi:10.3390/cells8030281)
Supplement: Supplementary file 1 [file cells-08-00281-s001.pdf]

Supplementary Figures

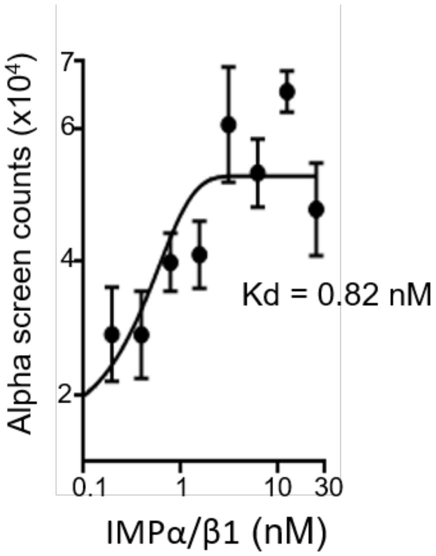

Fig. S1. High affinity recognition of DENV NS5 by the IMPα/β1 heterodimer. AlphaScreen technology was used to determine the dissociation constant (Kd) of binding of IMPα/β1 (prebound IMPα/β1 heterodimer with biotinylated IMPβ1) to His_6-DENV NS5 (30 nM). Data represent the mean +/- SD for triplicate wells from a single typical experiment, from a series of 2 independent experiments (see Table 1 column 1 for pooled data).

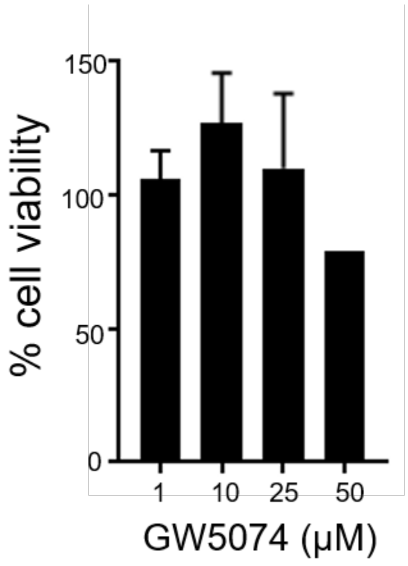

Fig. S2. Lack of toxicity of GW5074 at concentrations effective in inhibiting flavivirus. Cell viability was determined by addition of XTT reagent (Sigma-Aldrich) following compound treatments, as indicated. Cell survival is plotted relative to an untreated control. Data represent the mean +/- SD for duplicate wells from a single typical experiment, from a series of 2 independent experiments.

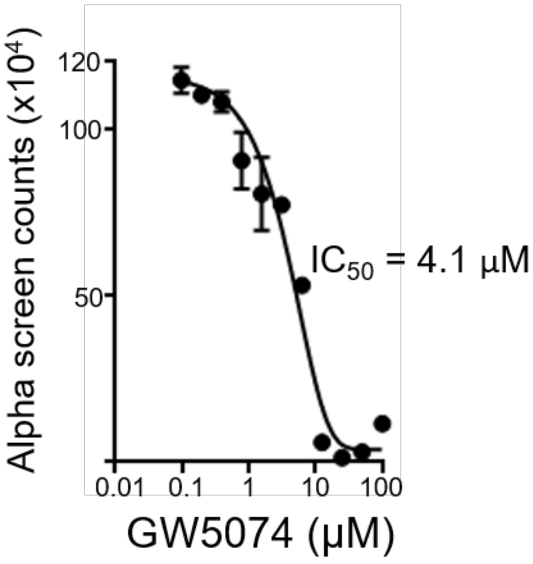

Fig. S3. GW5074 inhibits recognition of SV40 T-antigen by the IMPα/β1 heterodimer. AlphaScreen technology was used to determine the IC_{50} for inhibition by GW5074 of T-ag binding to IMPα/β1. Data represent the mean +/- SD for triplicate wells from a single typical experiment, from a series of 2 independent experiments.

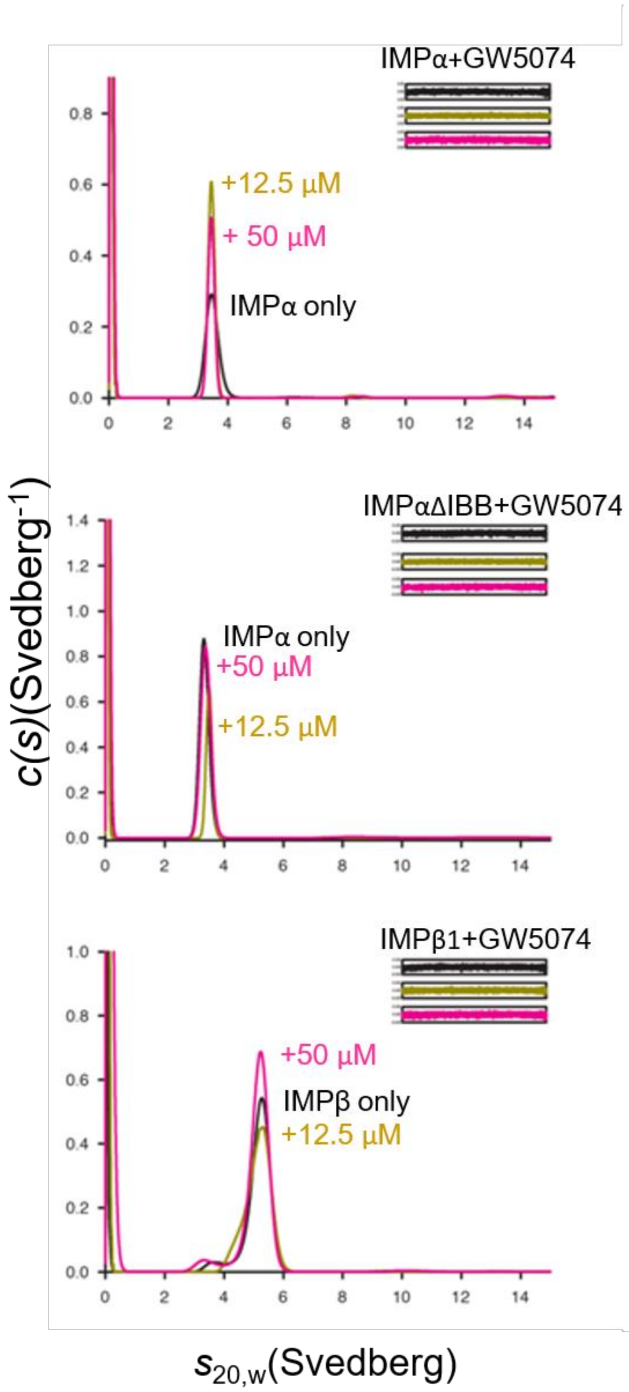

Fig. S4. GW5074 does not alter the sedimentation coefficients of IMPα, IMP$\alpha\Delta$IBB or IMPβ1. Sedimentation velocity analytical ultracentrifugation experiments were performed on purified recombinant IMPα, IMP$\alpha\Delta$IBB and IMPβ1, in the absence or presence of GW5074. The continuous sedimentation coefficient distribution [$(c)s$] was plotted as a function of $s_{20,w}$ for IMPs alone, in the presence of the indicated concentrations of GW5074. The residual plots are shown in insets. Results are from a single typical experiment, from a series of 2 independent experiments.

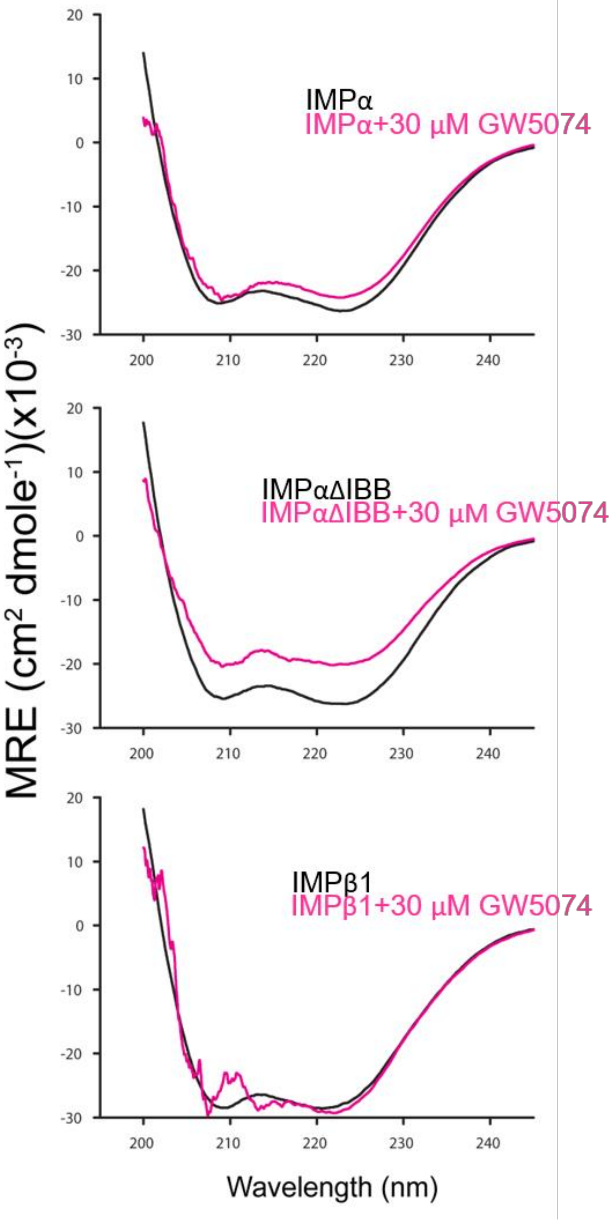

Fig. S5. CD spectra for IMPs in the absence and presence of GW5074. CD spectra were collected for IMPα, IMPαΔIBB and IMPβ1 in the absence or presence of 30 μM GW5074. The plots are representative of 2 independent experiments.

Supplementary Materials

Table S1. Hydrodynamic properties of recombinant IMP proteins.

Protein	Parameter			
	M_r[a]	M[b]	$s_{20,w}$[c]	f/f_0[d]
IMPα	58072	50316	3.5	2.4
IMPβ1	98557	106358	5.3	2.1
IMPα/β1	156629	155055	6.7	2.7

[a] Relative molecular weight calculated from the amino acid sequence.

[b] Molar mass determined from the ordinate maximum of $c(M)$ distribution best fits (data not shown).

[c] Standardized sedimentation coefficient taken from the ordinate maximum of the $c(s)$ distribution best fits (Fig. 2C).

[d] Frictional coefficient calculated from $s_{20,w}$ using the $\bar{v}$ method employing SEDNTERP.
